# Paralytic Deformities and Disabilities of the Feet

**Published:** 1919-11-01

**Authors:** 


### PARALYTIC DEFORMITIES AND DISABILITIES OF THE FEET.

### The Second of Two Post-Graduate Lectures.

In his second lecture in connection with the Fellowship of Medicine, delivered at the Royal Society of Medicine on Monday, September 22, Mr. William H. Trethowan dealt with the subject of paralytic deformities and dis bilities of the feet, and again he had a number of patients and plaster-casts to illustrate his thesis. We continue our summary of his teaching.

The deformities may involve either the upper neurons or the lower. They include poliomyelitis, neuritis, nerve injury, muscular dystrophy, hemiplegia, diplegia, and paraplegia. The majority of paralytic deformities of the feet are those due to involvement of the lower neurons. During the war there have been many cases due to gunshot wounds: some with flail feet, or with the popliteal or external groups of muscles gone, while many cases of dropped foot have been due to paralysis of the dorsiflexors and the peronei. The cases in which the internal group of muscles is involved have been but few. Neuritis in civil life has been mainly due to alcohol or to specific fevers, especially influenza, but the twisted deformities of the feet are most often seen in children.

Feet in which the disability is congenital are usually warm, normal hued, and bulky, not cold and blue and wasted as in acquired conditions.

As a rule, paralysis of the lower limb is flail-like, whereas in the upper it is stiff and of a spastic character. In the lower limb the reflexes are gone, while in the upper they are usually brisk.

In poliomyelitis, the disease is in the cord itself. Though in the early, acute stage the paralysis may be profound, yet at the end of six or twelve months the cells may have recovered with the exception of one group: for instance, the tibialis anticus may fail, and all the other muscles be good. Any combination of muscles may be affected, and it is only necessary to work out the resultant clinical condition to appreciate what resources are available to complete the treatment.

It must be remembered that these deformities are, in every case, the results of neglect. In the stage when the departure from the normal was but small, the lightest possible splint to preserve the foot in a steady square position would have sufficed to prevent the deformity, or if, at the end of six months, the muscles are not just as they should be, special attention can be given to the erring groups. When looking after an early case of poliomyelitis, the foot should be put and kept square with the leg. Then, if the tibialis is found to have recovered before the peronei, the foot should be turned slightly outwards, *i.e.*, erring in favour of the peronei. The best means of getting a paralysed muscle to recover is to keep the muscles short.

The best thing for a paralysed foot, after the acute stage has passed, is for the patient to get about. The lecturer was working in a large hospital in Sweden when there was an epidemic of poliomyelitis, and the practice was to get the patient up and about at the end of three months, the foot being put into that position in which the muscles which had not yet recovered were kept short. It has been found that the child who is struggling along is in the best circumstances for promoting recovery—it is much better than lying in bed. If the foot has been put in plaster, then into splints, then into light celluloid splints, and later special boots are fitted, the limb having been kept warm and treated with electricity during such treatment, one cannot expect rapid improvement after the lapse of, say, eighteen months, though for years slight improvement will go on in many.

These cases always present a primary deformity, and a secondary one. The first of these will depend on the particular group of muscles at fault, while the secondary deformity will be caused partly by gravity and partly by the unbalanced action of the remaining muscles. For example, the primary deformity may be equinus, the secondary one may be cavus.

If there be what appears like a settled deformity, what should be done? If there is no power in a limb—as in a case shown—the patient is much better without the joint, and hence an operation for the destruction of the joint is advisable. If half the normal power remains, efforts should be made to share that power between the two sides, *i.e.* by tendon transplantation. Before this, however, it is most important to observe an important orthopædic doctrine: *first passively correct the deformity.* In a case in which the peronei are gone, resulting in a dropped foot, one divides the sole tendons subcutaneously with a tenotomy knife, then restores the sole, with the heel in its proper position. It should be afterwards kept in plaster for a period of from three to six weeks. If dorsiflexion cannot then be performed, a Bayer's tenotomy should be done. Every effort should be made by the surgeon to see if there is any power of recovery left in the paralysed muscle. Many results of tendon transplantation are not satisfactory merely because the operator has not kept the limb fixed for a reasonable time to see if recovery has reached the utmost that is possible to the muscle concerned. It is most desirable, in tenotomising tendons, to keep away from the annular ligaments. One should always hesitate to tenotomise flexors: for them pressure splints or strapping are to be preferred. If one were forced to operate on them one would choose an open operation. Having put the foot into a proper position by tenotomy, physio-therapy should be carried out for some months.

Tarsectomy is of doubtful value: out of many hundreds of operations, the lecturer had not done

The previous article appeared on October 18 p. 51.

**Paralytic Deformities of the Feet**—(*continued*).

tarsectomy more than three times. In doing tendon transplantation, carry out sub-periosteal insertion of tendon: stitching tendons in with fishing-gut, running the tendon through the fat.

For the flail-like foot another procedure is carried out, namely, tenodesis, *i.e.*, cutting off tendons, and fastening the distal ends into bone. It can be done in cases in which the patient has to lift the knee higher than on the other side in order to prevent his foot catching on the ground. It is an alternative to instrumentation. The degree of shortening of the leg bears no relation to the extent of the foot paralysis. For drop-foot, it is well to put steel on the inside aspect of the foot, and provide a toe-raising spring in addition. In a spastic condition, contraction first occurs, and that proceeds to adaptive shortening and contracture. This develops more quickly in a flail paralysis: a spastic one is coaxable. Even spasm does not debar one from lengthening tendons, but it must be done with great care, and it should not be done on a spastic tendo Achillis, or the tendon will contract up the leg, and the resulting deformity will be very disappointing.

Spastic cases are much more liable than others to pressure sores and decubitus. If they are put into plaster use a large amount of wool and padding. If one is unfortunate enough to get a plaster sore excise it; it will not heal otherwise, but will be a source of much trouble.

Spastic cases require to be treated by re-education. Treat the antagonisers of the spastic muscles as you treat the paralysed muscles. In every case keep correction up for a few months before you assess the amount of permanent disability, and therefore before you proceed to do transplantation, arthrodesis, or tenodesis. Throughout it is important to remember that the foot should be kept soft and pliable; stiffness means disability.

